# Selected lifestyle factors as students transition from secondary school to university in Slovakia

**DOI:** 10.3389/fpubh.2024.1461989

**Published:** 2024-10-02

**Authors:** Alena Buková, Petra Tomková, Ivan Uher, Tatiana Kimáková, Ľuboš Vojtaško, Ferdinand Salonna

**Affiliations:** ^1^Institute of Physical Education and Sport, Pavol Jozef Šafárik University in Košice, Košice, Slovakia; ^2^Department of Public Health and Hygiene, Faculty of Medicine, Pavol Jozef Šafárik University in Košice, Košice, Slovakia; ^3^Institute of Languages, Social Sciences and Academic Sports, Technical University of Košice, Košice, Slovakia

**Keywords:** lifestyle changes, physical activity, sleep quality, dietary habits, alcohol consumption, tobacco use, transition to university, university students

## Abstract

**Background:**

The study aimed to evaluate self-perceived changes in lifestyle factors, particularly physical activity (PA), following the transition from secondary school to university. A secondary objective was to examine the relationship between lifestyle variables and weekly PA frequency. Key factors assessed included sleep quality, dietary habits, alcohol and tobacco consumption, and PA frequency.

**Methods:**

The study surveyed 1,665 first-year undergraduate students at Slovak public universities (mean age: 20.73 years, SD ±1.39) using adapted versions of Healthy Lifestyle Questionnaire (CEVS-II) and the Brief Pittsburgh Sleep Quality Index (B-PSQI). Data collection took place between December 2022 and February 2023.

**Results:**

Significant declines were observed in most lifestyle variables after students transitioned to university. The proportion of students engaging in irregular, or no PA increased from 37% before university to 46% during their studies (*p* < 0.01). Reports of tiredness upon waking also rose, from 52 to 64% (*p* < 0.01). Meal frequency decreased, particularly among males, with the percentage of students eating only 1–2 meals per day rising from 9 to 15% (*p* < 0.05). Conversely, the regularity of breakfast consumption increased, increasing from 65 to 73% (*p* < 0.01). No significant changes were observed in tobacco smoking habits.

**Conclusion:**

The findings highlight a notable reduction in PA and other lifestyle factors during the transition to university life. These results emphasize the need for targeted interventions to support healthy behaviors during this critical life phase.

## 1 Introduction

Entering university is a pivotal moment in young people’s lives, bringing significant changes across various aspects. While secondary education is marked by structured tasks and responsibilities overseen by parents and teachers, university students take on greater responsibility for their own health and habits. The first year of university is particularly critical for risk-taking behavior as students adapt to a new environment. However, this period also presents an opportunity to establish or modify healthy habits (1).

According to previous studies, it is estimated that around 50% of the global student population suffers from sleep problems (2). The primary barriers to achieving good sleep hygiene among university students include excessive use of technology (e.g., computers, cell phones, and exposure to light before bed), consumption of substances like caffeine, energy drinks, alcohol, and stimulants, as well as irregular study schedules and insufficient physical activity (3, 4). These factors significantly disrupt sleep patterns, leading to poor sleep quality and other related health issues (2).

Adolescence and young adulthood, including the transition to university, represent critical periods for the development of tobacco smoking habits. Over 30% of current smokers worldwide began smoking daily by the age of 16, with a significant portion starting between the ages of 15 and 24 (5).

Although heavy drinking among youth has been declining in European and other high-income countries since the 2000s, it remains prevalent at universities, largely because it plays a significant role in how students form social connections during a critical period—particularly in the early months of their first year (6). Many first-year students use alcohol as a tool to foster social bonds and integrate into key aspects of student life, such as student housing and social events organized by student societies (7).

In non-clinical populations, food addiction is more common among college students (24%) than the general population (20%) (8). College students are a recognized risk group for eating disorders, as the transition to college often leads to changes in eating habits. This period is typically marked by limited food variety, meal skipping, high-calorie snacking, and food choices driven by taste and pleasure (9).

There is strong evidence that regular physical activity (PA) could be a highly effective, non-pharmacological and non-invasive method of promoting health (10–12). A PA-supportive lifestyle is associated with a reduced risk of mortality and correlates with improved overall health (13, 14). A large number of epidemiological studies in recent decades have found that individuals who are more physically active have a lower incidence of cardiovascular disease and all-cause mortality compared to sedentary individuals (15). There are some important associations between individual lifestyle factors and PA. However, studies are inconclusive and often suggest conflicting findings.

Based on the above findings, the purpose of our study was to determine the lifestyle changes that occurred in students during the transition from secondary school to university, and to explore how certain factors were linked to students’ physical activity (PA) levels during both periods. We hypothesized that unhealthy behaviors would increase after students entered university.

## 2 Materials and methods

### 2.1 Participants and settings

The cohort for this cross-sectional study consisted of 1,665 first-year university students from all universities in Slovakia, who voluntarily participated. Of these participants, 488 (29.3%) were male, and 1,177 (70.7%) were female. The required sample size was calculated using a Sample Size Calculator to ensure statistical reliability, based on a population of 38,382 first-year bachelor’s degree students in Slovakia during the academic year 2022/2023. The confidence interval was set at 3, with a minimum sample size of 1,038 students.

Participants were between 18 and 24 years old, with an average age of 20.73 years (± 1.39). Males were significantly older than females, with a mean age of 20.95 years (± 1.43) compared to 20.64 years (± 1.36) for females (*p* < 0.001). Sociodemographic details such as age, gender, residence, and nationality are further outlined in Tables 1, 2.

**Table 1:** Basic somatic characteristics of the study participants.

Group	N	Age (years)	Weight (kg)	Height (cm)	BMI
All	1,665	20.73 ± 1.39	69.49 ± 11.7	174.3 ± 7.2	22.7 ± 3.43
Men	488	20.95 ± 1,43	78.9 ± 13,2	181.3 ± 7.9	23.96 ± 3.51
Women	1,177	20.64 ± 1.36	60.1 ± 10.3	167.3 ± 6.4	21.46 ± 3.35

**Table 2:** Basic demographic characteristics of the study participants (N).

Number of inhabitants	City over 50 thousand inhabitants	City under 50 thousand. Inhabitants	village
All	532	468	665
Men	168	134	186
Women	364	334	479
Focus of study	humanities and social sciences	technical sciences	medical sciences
All	816	381	468
Men	138	196	154
Women	678	185	314
Nationality	Czech	Slovak	Ukrainian
All	58	1,551	56
Men	27	447	14
Women	31	1,104	42

The inclusion criteria for the study were: being a first-year undergraduate student, aged between 18 and 24, and enrolled in any of the 16 public or 4 technical universities in Slovakia. Initially, 2,174 students were surveyed, but students from upper years (*n* = 386), those outside the 18–24 age range (*n* = 96), and students retaking their first year (*n* = 27) were excluded from the final sample (16, 17).

A questionnaire survey was used to conduct the research. Data collection started in December 2022 and was completed in February 2023. Female bachelor’s degree students were approached through Instagram and Facebook social networking sites, and the questionnaire was administered using the Google Form application. This way of completing a questionnaire is convenient and preferred by the younger generation. Another advantage of the online form is creating the questionnaire in such a way that requires all questions to be answered by the participants, without allowing for any questions to be left unfilled. The questionnaire included written information about the scientific significance of the study and information about the confidentiality of the answers. A precondition was the respondent’s willing consent to be involved in the study.

### 2.2 Applied questionnaires

Self-reported socio-demographic and health data (gender, date of birth, health status, residence, nationality, university, type of study), body height and weight date were collected. The Body Mass Index (BMI) was calculated as the body weight in kilograms divided by the square of height in meters and categorized as underweight (BMI <18.5 kg/m2), healthy weight (BMI 18.5 to 24.9 kg/m2), overweight (BMI 25 to 29.9 kg/m2), and obese (BMI > 30.0 kg/m2).

To assess lifestyle of university students we conducted a questionnaire survey. To measure balanced diet, respecting meal schedules, resting habits, tobacco consumption, alcohol consumption, use of other substances, and PA we used an adapted version the Healthy Lifestyle Questionnaire (CEVS-II). The internal consistency of the original CEVS-II is reported to be high across various dimensions of the questionnaire. For the different subscales Cronbach’s alpha values typically range from 0.70 to 0.85, indicating good reliability (18). However in our version the Cronbach’s alpha values ranged from borderline 0.58 for physical activity to good 0.88 for smoking.

To measure the sleep quality retrospectively over the previous month using self-report/recall we used an adapted version the Brief Pittsburgh Sleep Quality Index (B-PSQI) which is designed to assess overall sleep quality, sleep latency, sleep duration, and sleep disturbances. The original brief form has adequate internal consistency (Cronbach *α* = 0.79) (19). Our adaptation had lower but still satisfactory internal consistency (Cronbach α = 0.59).

Our adaptation was intended to assess the behavior of students during these two distinct periods. Specifically, we investigated individual lifestyle variables in the final year of secondary school (the last semester, excluding summer holidays) and upon entry to university (the first semester of study, excluding the exam period). As we aimed to monitor changes in sleep quality and quantity, as well as selected lifestyle factors, in relation to secondary school attendance, we supplemented the questionnaires with questions specifically designed to capture alterations in these variables before and during the first year of university.

The primary variable we related to lifestyle factors was the frequency of PA per week. We accepted any purposeful PA (cycling, running, swimming fitness, etc.) except for walking. These activities further excluded activities of a routine nature—housework, gardening, etc. Another condition was to perform a single PA for more than 10 min.

### 2.3 Statistical analysis

In terms of basic mathematical and statistical processing, in the so-called univariate descriptive statistics, parameters such as absolute and relative frequency were employed for the statistical description of variables.

Non-parametric tests were used for the analysis, due to the lack of normality of distribution of the examined variables or due to their ordinal nature. The normality of the distributions was assessed using the Shapiro–Wilk W test. The Pearson chi-square test was used to compare the variables in the two groups. The Wilcoxon pairwise order test was used to compare the results over two time periods in the same group.

Statistical analysis was conducted using the IBM SPSS Statistics software version 29, and the statistical significance of the results obtained was determined at a significance level of *α* = 0.05.

## 3 Results

BMI of the participants ranged from 13.41 to 44.98 kg/m2. The average BMI of the respondents was 22.2 kg/m2 ± 3.63 kg/m2. Men had significantly higher BMI compared to women (*p* < 0.001; Table 3).

**Table 3:** BMI of the study participants.

BMI [kg/m^2^]	Basic descriptive statistics							
	N	Mean	Median	Min.	Max.	Q I	Q III	SD
Women	1,177	21.47	20.70	13.41	44.98	19.23	22.94	3.42
Men	488	23.96	23.52	16.80	41.97	21.74	25.64	3.51
All	1,665	22.20	21.48	13.41	44.98	19.61	24.17	3.63
*p*	Z = −14.68, *p* < 0.001							

Z, value of the Mann–Whitney U test; *p*, test probability index.

Most of the subjects were of normal weight (70.3% of the total). Normal weight and underweight were more common in women, while overweight and obesity were more common in men. The difference in body build between men and women was statistically significant (*p* < 0.001; Table 4).

**Table 4:** BMI ranges of the study participants.

BMI	Women		Men		All	
	N	%	N	%	N	%
Below average	173	14.7%	13	2.7%	186	11,2%
Average	845	71.8%	325	66.6%	1,170	70.3%
Overweight	126	10.7%	123	25.2%	249	15.0%
Obesity	33	2.8%	27	5.5%	60	3.6%
All	1,177	100.0%	488	100.0%	1,665	100.0%
*p*	χ^2^(3) = 101.67, *p* < 0.001					

χ^2^, value of Pearson’s chi-square test; *p*, test probability index.

Compared to the period of study at secondary school, the current body weight of 33.3% of the respondents was higher, 23.1% lower, while 37.8% of the respondents reported no change, and 5.9% of them were unable to assess the change. Men (39.8% vs. 30.6%) were more likely to report higher current body weight. In contrast, women more often declared having a currently lower (25.5% vs. 17.2%) or the same (38.1% vs. 37.1%) body weight. Gender differences were statistically significant (*p* < 0.001; Table 5).

**Table 5:** Weight change compared to secondary school.

	Women		Men		All	
	N	%	N	%	N	%
Higher	360	30.6%	194	39.8%	554	33.3%
Lower	300	25.5%	84	17.2%	384	23.1%
No change	448	38.1%	181	37.1%	629	37.8%
Hard to tell	69	5.9%	29	5.9%	98	5.9%
All	1,177	100.0%	488	100.0%	1,665	100.0%
*p*	χ^2^(3) = 19.05, *p* < 0.001					

χ^2^, value of Pearson’s chi-square test; *p*, test probability index.

The change in weight gain also differed between men and women (*p* = 0.046). On average, men gained more weight than women. However, the most common weight gain was between 0 and 3 kg (53.5%), less common was 4–5 kg (29.8%) or more than 6 kg (16.8%; Table 6).

**Table 6:** Change in weight compared to secondary school—increase.

	Women		Men		All	
	N	%	N	%	N	%
Increase by 0–3 kg	257	55.4%	126	50.0%	383	53.5%
Increase by 4–5 kg	141	30.4%	72	28.6%	213	29.8%
Increase by 6 kg and more	66	14.2%	54	21.4%	120	16.8%
All	464	100.0%	252	100.0%	716	100.0%
*p*	χ^2^(2) = 6.12, *p* = 0.046					

χ^2^, value of Pearson’s chi-square test; *p*, test probability index.

The negative change did not differ between men and women (*p* = 0.537). The weight loss of the students was usually no more than 3 kg (62.7%; Table 7).

**Table 7:** Change in weight compared to secondary school—decrease.

	Women		Men		All	
	N	%	N	%	N	%
Lower by 0–3 kg	192	61.9%	63	65.0%	255	62.7%
Lower by 4–5 kg	65	21.0%	22	22.7%	87	21.4%
Lower by 6 kg and more	53	17.1%	12	12.4%	65	16.0%
All	310	100.0%	97	100.0%	407	100.0%
*p*	χ^2^(2) = 1.24, *p* = 0.537					

χ^2^, value of Pearson’s chi-square test; *p*, test probability index.

A significant difference between the lifestyle variables examined before and after entering university was found for most variables (Table 8). Although there was no change in the time at which students fell asleep, arriving at university increased the number of students falling asleep after 10 pm and decreased the number of students falling asleep before 10 pm. The number of students falling asleep within 30 min decreased and the number of students falling asleep for more than 30 or 60 min increased. The universities are dominated by students who get up after 8 am, which is to be expected given the nature of university study. We found no difference in the variable number of hours of sleep for males compared to secondary school. The difference in substance use (alcohol, smoking) between secondary school and university is only significant for smoking among female students.

**Table 8:** The relationship between lifestyle variables before and during university (in %).

	Before going to university				First semester of study						*p*	
											Z	signif.
**Sleep duration**	before 8 p.m.	8–9 pm.	9–10 p.m.	after 10 p.m.		before 8 p.m.	8–9 p.m.	9–10 p.m.	after 10 p.m.			
Men	0.82	4.51	21.52	73.16		0.61	1.64	13.73	84.02		−6.111	*p* < 0.01
Women	0.34	5.52	27.36	66.78		0.34	2.80	14.70	82.16		−10.568	*p* < 0.01
All	0.48	5.23	25.65	68.65		0.42	2.46	14.41	82.70		−12.198	*p* < 0.01
**Falling asleep**	up to 10 min.	10–30 min.	30–60 min.	more than 60 min.		up to 10 min.	10–30 min.	30–60 min.	more than 60 min.			
Men	34.84	47.95	15.16	2.05		27.87	44.88	18.85	8.40		−7.325	*p* < 0.01
Women	31.10	47.15	17.67	4.08		24.04	39.00	24.89	12.06		−12.393	*p* < 0.01
All	32.19	47.39	16.94	3.48		25.17	40.72	23.12	10.99		−14.383	*p* < 0.01
**Waking up**	before 6 a.m.	6–7 a.m.	7–8 a.m.	after 8 a.m.		before 6 a.m.	6–7 a.m.	7–8 a.m.	after 8 a.m.			
Men	13.32	37.91	29.92	18.85		9.22	21.11	34.84	34.84		−8.930	*p* < 0.01
Women	12.57	42.31	27.44	17.67		6.63	20.56	35.26	37.55		−16.224	*p* < 0.01
All	12.79	41.02	28.17	18.02		7.39	20.72	35.14	36.76		−18.516	*p* < 0.01
**Number of hours of sleep**	6 h and less	6–7 h	7–8 h	8 h. and more		6 h and less	6–7 h	7–8 h	8 h. and more			
Men	9.43	34.43	42.01	14.14		12.09	28.48	41.19	18.24		−1.224	*p* = 0.221
Women	10.37	34.49	42.57	12.57		12.74	30.08	35.68	21.50		−2.856	*p* < 0.05
All	10.09	34.47	42.40	13.03		12.55	29.61	37.30	20.54		−3.093	*p* < 0.05
**Morning tiredness upon waking up**	yes	no				yes	no					
Men	47.95	52.05				58.40	41.60				−4.715	*p* < 0.01
Women	53.87	46.13				66.95	33.05				−7.643	*p* < 0.01
All	52.13	47.87				64.44	35.56				−8.964	*p* < 0.01
**Frequency of alcohol consumption**	1–2 x/w	3 and more/w	1–3 x/ month	never/ occasionally		1–2 x/w	3 and more/w	1–3 x/ month	never/ occasionally			
Men	15.98	3.07	35.86	45.08		14.14	4.30	31.15	50.41		−1.350	*p* = 0.177
Women	10.20	1.78	35.17	52.85		9.26	2.29	30.84	57.60		−2.542	*p* < 0.05
All	11.89	2.16	35.38	50.57		10.69	2.88	30.93	55.50		−2.837	*p* < 0.01
**Smoking**	1–2 x/w	3 and more/w	daily	never/ occasionally		1–2 x/w	3 and more/w	daily	never/ occasionally			
Men	1.64	11.27	0.82	86.27	2.46	10.66	1.23		85.66		−0.151	*p* = 0.880
Women	1.95	11.47	0.25	86.32	2.12	10.54	0.42		86.92		−1.768	*p* = 0.077
All	1.86	11.41	0.42	86.31	2.22	10.57	0.66		86.55		−1.560	*p* = 0.119
**Meal frequency**	1–2 x /day	3–4 x /day	5–6 x /day			1–2 x /day	3–4 x /day	5–6 x /day				
Men	9.22	71.11	19.67			14.75	64.96	20.29			−2.050	*p* < 0.05
Women	12.40	68.05	19.54			16.40	62.11	21.50			−1.107	*p* = 0.268
All	11.47	68.95	19.58			15.92	62.94	21.14			−1.948	*p* = 0.051
**Regularity of breakfast**	yes/ mostly yes	no/ mostly no				yes/ mostly yes	no/ mostly no					
Men	68.03	31.97				71.93	28.07				−2.165	*p* < 0.05
Women	63.81	36.19				73.32	26.68				−6.403	*p* < 0.01
All	65.05	34.95				72.91	27.09				−6.694	*p* < 0.01
**Daily intake of fruits and vegetables**	yes/ mostly yes	no/ mostly no				yes/ mostly yes	no/ mostly no					
Men	67.01	32.99				66.80	33.20				−0.124	*p* = 0.901
Women	73.07	26.93				74.51	25.49				−1.230	*p* < 0.01
All	71.29	28.71				72.25	27.75				−1.00	*p* < 0.01
**Frequency of PA per week**	1–2 x/w.	3 and more/w	irregularly	none PA		1–2 x/w.	3 and more/w	irregularly		no PA		
Men	30.53	45.08	20.29	4.10		30.33	29.71	30.74	9.22		−7.464	*p* < 0.01
Women	31.01	27.19	33.05	8.75		29.14	22.34	35.51	13.00		−5.021	*p* < 0.01
All	30.87	32.43	29.31	7.39		29.49	24.50	34.11	11.89		−8.288	*p* < 0.01

The results are also ambiguous when it comes to diet. We found no difference in the frequency of eating for female students compared to secondary school, and in the daily consumption of fruit and vegetables for males. The main difference between secondary school and university is observed in the implementation of PA. At university, there was a significant increase in the number of students who engaged in PA irregularly or not at all. The decrease is particularly noticeable among women.

In relation to gender (Table 9), we found statistically significant differences both before and during university in only 4 variables—morning tiredness upon waking up, frequency of alcohol consumption, daily intake of fruits and vegetables, and frequency of PA per week.

**Table 9:** Gender-related lifestyle factors.

	Before going to university		First semester of study	
	χ^2^	significance	χ^2^	significance
Sleep duration	8.965	*p* < 0.05	2.888	*p* = 0.409
Falling asleep	6.903	*p* = 0.075	14.590	*p* < 0.01
Waking up	2.796	*p* = 0.424	3.903	*p* = 0.272
Number of hrs. of sleep	0.976	*p* = 0.807	4.998	*p* = 0.172
Morning tiredness upon waking up	4.837	*p* < 0.05	11.001	*p* < 0.01
Frequency of alcohol consumption	16.530	*p* < 0.01	15.739	*p* < 0.01
Smoking	4.299	*p* = 0.507	5.135	*p* = 0.400
Meal frequency	3.516	*p* = 0.172	1.270	*p* = 0.530
Regularity of breakfast	2.710	*p* = 0.100	0.340	*p* = 0.560
Daily intake of fruits and vegetables	6.188	*p* < 0.05	10.224	*p* < 0.05
Frequency of PA per week	63.366	*p* < 0.01	14.254	*p* < 0.01

Regarding individual lifestyle variables in relation to PA implementation (Table 10), we observed a significant relationship for a number of variables during university and for most variables reported prior to entering university.

**Table 10:** The relationship between PA and other lifestyle factors.

	Before going to university		First semester of study	
	χ^2^	significance	χ^2^	significance
Sleep duration	31.253	*p* < 0.01	23.64	*p* < 0.01
Falling asleep	9.936	0.644	12.29	0.20
Waking up	18.865	*p*< 0.05	14.29	0.11
Number of hrs. of sleep	8.308	0.503	16.08	*p* < 0.05
Morning tiredness upon waking up	14.302	*p* < 0.05	23.03	*p* < 0.01
Frequency of alcohol consumption	14.085	0.119	30.08	*p* < 0.01
Smoking	29.302	0.15	31.18	*p* < 0.01
Meal frequency	36.249	*p* < 0.01	61.94	*p* < 0.01
Regularity of breakfast	34.004	*p* < 0.01	28.00	*p* < 0.01
Daily intake of fruits and vegetables	50.161	*p* < 0.01	38.99	*p* < 0.01

## 4 Discussion

The aim of this study was to examine the extent to which changes in individual lifestyle factors occur with the transition from secondary school to university. A secondary aim was to examine the association between the individual factors and the frequency of PA per week. The results of our research suggest changes in several lifestyle factors upon transition from secondary school to university. We confirmed negative changes in sleep and dietary regimen; the greatest difference between secondary school and university study was found in the frequency of PA per week. In the latter, we observed a higher number of students exercising regularly 1–2 times per week, whereas after entering university, the number of students exercising only irregularly or not at all increased significantly. According to the standards of daily PA developed by the WHO, university students can be classified as a group of physically inactive persons with a strong tendency toward a sedentary lifestyle, which is in sharp contrast to their openly declared attitude toward PA (20). Longitudinal studies observing adolescents have shown a 24% decrease in PA, with the largest decrease occurring in the first year of college (21). In Europe, physical inactivity reaches 27% in adults aged 18 years and over, including the university population, with an even higher percentage of inactive adults in Slovakia (34.9%) (22). A review of 298 studies from 146 countries by Guthold et al. (23) found a high prevalence of inactive secondary school students aged 11–17 years (81%). Based on our research, we are consistent with Deliens et al. (24), who reported that this prevalence is highly likely to continue at university, as at this period undergraduates are often making autonomous lifestyle choices (e.g., behavior, eating) for the first time.

The transition to college or university is considered a critical period for students in forming habits. This is primarily due to changes in diet, but also in sleep, PA and increased alcohol intake as a potentially new legal experience and its availability without overt parental control. This was confirmed in our study for both sexes, except for the risk behaviors of alcohol intake in males and smoking in both sexes. Yet, some studies point to increased alcohol consumption and smoking during the transition to early adulthood (21). Research shows (25) that up to 20–40% of students engage in binge drinking. A systematic review of the determinants of risk behaviors among university students was conducted in 2017 by Arsandaux et al. (26). The authors reviewed 111 articles on the use of different substances (alcohol, tobacco, drugs). The majority of studies on alcohol is related to the frequency of consumption (*n* = 26), alcohol abuse (10) and binge drinking (*n* = 7). Several other studies dealt with smoking (n = 10), of which 9 studies investigated the relationship between self-esteem and smoking. Salgado García et al. (27) in a study of college students (*n* = 288) found an association between alcohol consumption and smoking and several other factors: different life domain stressors, family, lack of social support, and childhood stressors. Miramontes et al. (28) who surveyed Spanish first-year undergraduate students over 3 years found increased at-risk alcohol use among students who lived away from home, increased risk of binge drinking, and heavy episodic drinking among students who started earlier. The authors partially confirmed a relationship between increased risk of alcohol consumption and the mother’s education.

Lifestyle risk factors, which include smoking, alcohol abuse, unhealthy diet and physical inactivity, cluster under certain socio-demographic groups, including gender. Several studies have reported higher levels of physical inactivity in women compared to men and across all age groups (29, 30). Our study confirms this fact. Gender differences were also evident in alcohol consumption, which is consistent with some studies reporting higher alcohol consumption among men (31, 32). Regarding gender differences in dietary habits, we found no gender differences except for fruit and vegetable intake, in both secondary school and university. In general, women are more likely to adhere to a healthy diet and maintain healthy habits, while men are less likely to seek counseling on nutrition and health improvement (33, 34). Female students are also more interested in changing their eating habits than males. On the other hand, women are in most cases dissatisfied with their physique, and this combined with the influence of their environment and television advertising, may lead to eating disorders and poor eating habits, various diets, dietary modifications or even skipping meals.

In relation to the implementation of PA and individual lifestyle factors, we reached inconsistent findings. We found significant differences in most examined variables upon entering university, but not in secondary school. We found significant relationships in all items studied as regards dietary regimen. Eating habits, according to some authors, are mostly not directly related to the level of PA, which has been supported by several studies (35–37). However, most of the existing research on the relationship between PA and dietary habits deals with the pediatric population and, as reported by Sisay (35), the studies do not indicate how different levels of PA affect eating habits. Physical fitness and eating habits of students are closely linked to their attitudes toward health promotion and disease prevention (35, 38).

We found a consistent association, both after and before starting university, only between PA and bedtime and morning tiredness after waking up. Atkinson and Davemme (39) report several links between sleep and PA. They stress that an adequate transition period between PA and the onset of sleep may well be important to ensure good sleep. However, further research is needed to determine the optimal amount and timing of PA to reduce sleep-related problems. Short sleep duration appears to negatively affect engagement in PA (40). The authors found that late bedtimes were associated with frequent sitting and less time spent doing moderate PA. Flueckiger et al. (41) investigated the impact of PA and sleep quality on learning. Even average sleep quality had a significant effect on achieving a learning goal, but the study did not find a significant relationship between PA and learning. A connection between sleep duration, sedentary behavior, and PA was found by Chastin et al. (42). However, the authors did not find a direct relationship between sleep and PA.

With regard to associations between PA and risky behaviors (alcohol and smoking), we found such associations only among university students. In a study conducted by Henchoz et al. (43), PA that included sports and daily activities during work and leisure time was positively correlated with at-risk alcohol consumption. As reported by the authors, athletes may be encouraged to drink excessively compared to non-athletes due to peer pressure. This may be part of social norms or a form of compensation for the stress and anxiety in sport, the latter being more likely in competitive athletes.

Despite the claim by several authors that students tend to gain weight during the transition from secondary school to university (44–46), our study did not confirm such a finding. However, the subjective reporting of body weight by the participating students and their subjective reporting of weight change with the onset of university may be considered a drawback of our survey in this regard.

To build on the findings of the study and translate these insights into actionable strategies, it is essential to consider practical interventions that universities can implement to promote healthier behaviors among students.

To address the decline in physical activity (PA) among university students, institutions should implement accessible fitness programs such as group workouts or intramural sports to encourage regular exercise. Alongside this, sleep management workshops and resources are essential, given the increase in reported tiredness upon waking, to help students improve sleep quality (3). Dietary support is also crucial, with universities offering affordable, healthy meal options and promoting regular eating habits, particularly as meal frequency has decreased among students (9). Mental health resources, including stress management programs and counseling services, are necessary to address the broader lifestyle declines linked to the transition to university life (47).

To effectively introduce health behavior interventions, universities should incorporate health-focused sessions into pre-university orientation programs, introducing students to wellness resources such as gyms, counseling, and nutrition services. Peer mentorship programs can further support students in adopting healthy habits. On-campus health services should be easily accessible and include counseling, nutrition, and workshops on healthy living. Health campaigns, delivered through social media and campus events such as wellness fairs and “Healthy Eating Days,” can engage students in maintaining positive behaviors. Optional physical education courses, flexible gym access, and infrastructure like walking paths should be promoted to support active lifestyles.

Additionally, providing healthy dining options and organizing alcohol-free social events, while supporting student societies in integrating health-focused activities, can help foster a balanced and healthy campus environment. These early interventions are essential in helping students establish lasting healthy habits during their critical transition to university life (1).

In the context of the conclusions, the limitations of this study must be taken into account when interpreting the results. The present study was cross-sectional and no longitudinal data were available to assess differences in the lifestyle factors studied. The subjective self-assessment of individual lifestyle factors and the use of a retrospective design can be considered the disadvantages of this study. Based on these facts, we cannot rule out the possibility that recollections of individual lifestyle factors from the last 6 months, with the exception of summer vacation and the first 3 months at university, may have been subject to recall bias. Another limitation concerns the selection of the sample. The sample consisted of voluntary participants. Since no random or stratified sampling techniques were used, we did not record the refusal rate; it is not known to what extent it was truly representative of the population. One particular drawback of the study is the disproportion between males and females, which may not be representative of the male first-year university population.

The strength of this study lies in the large size of the study sample. The sample comprises first-year university students from Slovak universities and is representative with respect to demographic factors. To the best of our knowledge, no research on individual lifestyle factors, including physical activity, has been conducted on such a sample of undergraduate students in Slovakia. Our study thus makes a meaningful contribution to the literature on this topic.

## 5 Conclusion

The results of the analysis of the research conducted among Slovak university students indicate changes in most of the observed lifestyle factors at transitioning from secondary school to university. The results also proved an association between the selected lifestyle variables and physical activity. On the contrary, gender differences in the variables studied were minimal and ambiguous. The assessment of individual lifestyle factors of university students appears to be important, especially in the first year of study. This period of life is the final stage of formal education, allowing for the formation of healthy habits. This also applies to influencing the health of undergraduates and preventing a range of health problems. This study supports the importance of implementing PA at university. Universities and other institutions of higher education are well suited in terms of providing programs that promote healthy behaviors, improve health literacy, and prevent the onset of civilizational diseases. These outcomes can be reflected in the quality of life in later years. Many internationally renowned universities are becoming increasingly aware of the need to promote student health, including the promotion of physical activity and a healthy lifestyle. University education should go beyond specialization, and higher education should further cultivate people not only intellectually, but also in terms of their overall personality and physical well-being.

## Data Availability

The raw data supporting the conclusions of this article will be made available by the authors, without undue reservation.
